# Non-invasive transcranial volumetric ultrasound localization microscopy of the rat brain with continuous, high volume-rate acquisition

**DOI:** 10.7150/thno.79189

**Published:** 2023-02-05

**Authors:** Jacob R. McCall, Francisco Santibanez, Hatim Belgharbi, Gianmarco F. Pinton, Paul A. Dayton

**Affiliations:** 1 The Joint Department of Biomedical Engineering, UNC Chapel Hill and NC State University; 2 Electrical and Computer Engineering, NC State University

**Keywords:** 3-D ultrasound, transducer, microvascular imaging, brain, super resolution

## Abstract

***Rationale:*** Structure and function of the microvasculature provides critical information about disease state, can be used to identify local regions of pathology, and has been shown to be an indicator of response to therapy. Improved methods of assessing the microvasculature with non-invasive imaging modalities such as ultrasound will have an impact in biomedical theranostics. Ultrasound localization microscopy (ULM) is a new technology which allows processing of ultrasound data for visualization of microvasculature at a resolution better than allowed by acoustic diffraction with traditional ultrasound systems. Previous application of this modality in brain imaging has required the use of invasive procedures, such as a craniotomy, skull-thinning, or scalp removal, all of which are not feasible for the purpose of longitudinal studies.

***Methods:*** The impact of ultrasound localization microscopy is expanded using a 1024 channel matrix array ultrasonic transducer, four synchronized programmable ultrasound systems with customized high-performance hardware and software, and high-performance GPUs for processing. The potential of the imaging hardware and processing approaches are demonstrated in-vivo.

*Results*: Our unique implementation allows asynchronous acquisition and data transfer for uninterrupted data collection at an ultra-high fixed frame rate. Using these methods, the vasculature was imaged using 100,000 volumes continuously at a volume acquisition rate of 500 volumes per second. With ULM, we achieved a resolution of 31 *µ*m, which is a resolution improvement on conventional ultrasound imaging by nearly a factor of ten, in 3-D. This was accomplished while imaging through the intact skull with no scalp removal, which demonstrates the utility of this method for longitudinal studies.

***Conclusions:*** The results demonstrate new capabilities to rapidly image and analyze complex vascular networks in 3-D volume space for structural and functional imaging in disease assessment, targeted therapeutic delivery, monitoring response to therapy, and other theranostic applications.

## Introduction

The vascular system is composed of a complex network of veins and arteries that carry oxygen- and nutrient-rich blood to capillaries, which allow the nutrients to pass into tissue cells, and then depleted blood to return to the heart via the veins. A vast number of pathologies are directly or indirectly associated with abnormal development or dysfunction of the vascular system, ranging from cancer, stroke, and dementia. It has been shown in cancer models, for example, that high vessel density in breast tumors is an independent predictor of metastasis 1,2. It was further shown that tumor malignancy can be characterized solely by the appearance of its vasculature 3. Gessner et al. and Shelton et al. have both demonstrated not only that these vascular differences are associated with tumor presence, but are also quantifiable 4,5 using acoustic angiography. Shelton et al. later showed in a reader study that a qualitative discrimination between healthy and tumor tissue can be made 6. The expression of disease in the vasculature is not limited to cancer, however. Many authors have recognized a decrease in cerebral blood flow in early stages of Alzheimer's disease and dementia 7-9. A large study of Dutch adults demonstrated that retinal microvascular abnormalities are predictive of cardiovascular mortality 10. Gianturco et al. identify a link between cardiovascular disease and systemic autoimmunity and calls for a “crucial need” for systems that can detect abnormality in microcirculation for the purpose of early diagnosis 11. Landers-Ramos et al. noted that skeletal muscle capillarization is reduced in sedentary adults and may contribute to physical and metabolic impairments 12. Medical imaging of the pre-cerebral and cerebral vasculature is a primary tool for the diagnosis of ischemic stroke 13. Computed tomography angiography (CTA) is highly sensitive and can readily detect intracranial arterial occlusions and stenoses in acute ischemic stroke 13,14. The radioactive contrast used for CTA, however, poses a risk of radio-contrast nephropathy 13.

The utility of a vascular imaging modality for monitoring response for therapy is of great value. Using a photoacoustic microvascular imaging technique in a mouse ear xenograft model, Zhou et al. demonstrated the ability to observe tumor vascular changes in response to treatment with an anti-angiogenic agent 15. Kasoji et al. demonstrated that tumor recurrence after radiation therapy could be detected earlier by using a vascular density metric compared to using a measure of tumor volume alone 16. Bullitt et al. have used magnetic resonance angiography (MRA) to draw significant conclusions on the response of glioblastoma multiforme to tumor resection and the treatment of anti-angiogenic agents by observing the geometry of the vasculature in a human brain 17.

Many of these methods for vascular imaging have significant drawbacks. Optical resolution photoacoustic methods can produce high resolution images but are limited to penetration depths up to a few millimeters 15,18. High-resolution MRA is capable of reconstructing microvasculature with voxel sizes as low as 52 *µ*m in a mouse brain, but with a long scan time up to 45 minutes 19. Microscopic computed tomography (micro-CT) has been used for angiography in mice via the use of contrast agents and has been shown to resolve vessels down to 50 *µ*m, but this method exposes the patient to radiation, is expensive, and may not provide desired hemodynamic information 20,21. Volumetric reconstruction of microvasculature using micro-CT also requires a scan time between 30 and 120 minutes 22,23. Ultrasonic Doppler imaging can be used to visualize flow intensity using power Doppler and directionality using color Doppler. Demené et al. demonstrated the ability to resolve large 3-D vascular networks down to 100 *µ*m in a rodent brain 24. Acoustic angiography is another ultrasonic tool for visualizing vascular networks that works by receiving microbubble (MB) signal at multiple harmonics above the transmit frequency. This method has been used for quantifying vessel tortuosity in the tumor angiogenesis 5. Acoustic angiography, however, is limited by the lack of available transducers with sufficiently wide bandwidth, and both acoustic angiography and power Doppler are constrained to a diffraction-limited resolution. Optical methods, such as two-photon vascular imaging, are able to resolve vasculature with high resolution, but are limited to penetration depths on the order of hundreds of micrometers 25.

Another method for microvascular imaging is ultrasound localization microscopy (ULM), which burgeoned from advancements in contrast enhanced ultrasound (CEUS) 26 and ultrafast ultrasound imaging 27. This method, which is able to resolve vasculature up to ten times the diffraction limit, has repeatedly demonstrated a remarkable ability to resolve complex vascular networks *in vivo* 28-31 and in humans 32,33. By imaging at hundreds to thousands of frames per second and by localizing and tracking MB signals, this method is not only able to resolve vascular structure, but is also able to resolve even slow vascular flow *in vivo* with values that are consistent with the literature 28,34,35. Demené et al. have demonstrated that ULM can provide vascular flow information with enough accuracy to detect an aneurysm through the skull in the human brain 32.

Until recently, this method has been limited to two-dimensional visualization, which is not ideal for visualizing complex three-dimensional networks of vasculature. Lately, the use of 2-D matrix arrays for ULM has been emerging in the literature. Chavignon et al. demonstrated transcranial ULM in a rat brain using a single Verasonics (Verasonics, Inc., Kirkland, WA, USA) Vantage system and a 7.8 MHz mutliplexed 32×32 matrix probe 36. This group was able to resolve vessels down to 31 *µ*m and compare their imaging technique with micro-CT. However, they noted that the multiplexed 256 channel system limited the number of transmitted plane-waves possible without decreasing the volume rate. As a result, a total scan time of 7 minutes was required to obtain a total of 100k B-mode images that were used for reconstruction. Demeulenaere et al. showed a transcranial, ULM acquisition of a whole mouse brain using a 4-Vantage system with a 7.8 MHz 32×32 matrix array 37. They demonstrated a registration of the detailed vasculature with the Allen mouse brain atlas 38 and quantified the flow rate in each vessel as a function of the radius. With their setup, this group was able to acquire 16 plane-waves at a volume rate of 750 volumes per second (vps). However, due to data transfer time, they were limited to 400 ms acquisitions with 3 s breaks in between acquisition blocks. As a result, the total scan time required to obtain a total of 90k volumes was 15 minutes. Additionally, both groups reported that the skin atop the skull was removed on some animals to avoid image degradation caused by unshaved hair or bubbles trapped on the skin.

In such settings where little motion is present, scan times on the order of 7 to 15 minutes may not present a significant challenge. However, acquisition in a clinical setting is not so ideal. Due to the prevalence of motion, more bubble localizations may need to be acquired in order to fully resolve vasculature, and this generally implies that more images must be collected in that time. In these scenarios, it's of great importance that there are no barriers to fast image acquisition. Huang et al. have demonstrated the ability to reconstruct microvasculature in the clinic in 2-D with short acquisition times using a high MB dose and a MB separation filtering step 33. This method hinges on fast acquisition of a few thousand frames on the order of seconds, which may not be possible with intermittent pauses in the 3-D acquisition. Huang et al. also utilize motion correction in order to maximize the number of useful frames 33. This implies the need for fast scan times so that proper motion correction can be applied on a sub-pixel scale. Acquisition at too low of a frame-rate may decrease image quality if the slow-time sampling rate is not sufficient to capture voluntary or involuntary patient motion.

In this work, we demonstrate our implemented method for continuous, ultrafast ultrasound acquisition at a fixed volume rate using a 1024-channel system consisting of four programmable ultrasound systems (Verasonics Vantage 256). The large quantity and rate of data generation required for volumetric imaging can be a challenge for acquisition time and maintaining consistent frame-rates. We utilized the Vantage buffer system in a unique way that separates the data acquisition from data transfer processes. This asynchronous acquisition and transfer of data at high volume rates removes the need for pausing during the acquisition, which reduces the total experiment time by up to a factor of five compared to the current state of the art in volumetric ULM. Using this method, we demonstrate a completely surgically non-invasive, i.e. transcutaneal and transcranial, fully volumetric acquisition in a rat brain in a total of 200 seconds. We also use Fourier shell correlation on a single volume 39,40 to show the ability to resolve vessels down to 31 *µ*m. We also demonstrate the visualization of various regions of the brain by registering the volumetric ULM image with a magnetic resonance imaging (MRI)-based neuroanatomical atlas of a Fischer 344 rat 41.

## Methods

MBs were formulated in-house as described by Tsuruta et al. and Kierski et al. 42,43. These bubbles were characterized using an Accusizer Nano FX (Entegris, Billerica, MA). The vial was measured to have a stock concentration of 2.7e10 MB/mL with a mean size of 1.6 *µ*m and a standard deviation of 1.4 *µ*m. The solution was diluted to a concentration of 3.3 MB/mL/g, based on the measured weight of the animal, and infused into the animal at a rate of 6 *µ*L/min. The use of a stir bar to prevent sedimentation of the MB solution in the syringe was not necessary since the scan time was very low.

**Figure 1:**
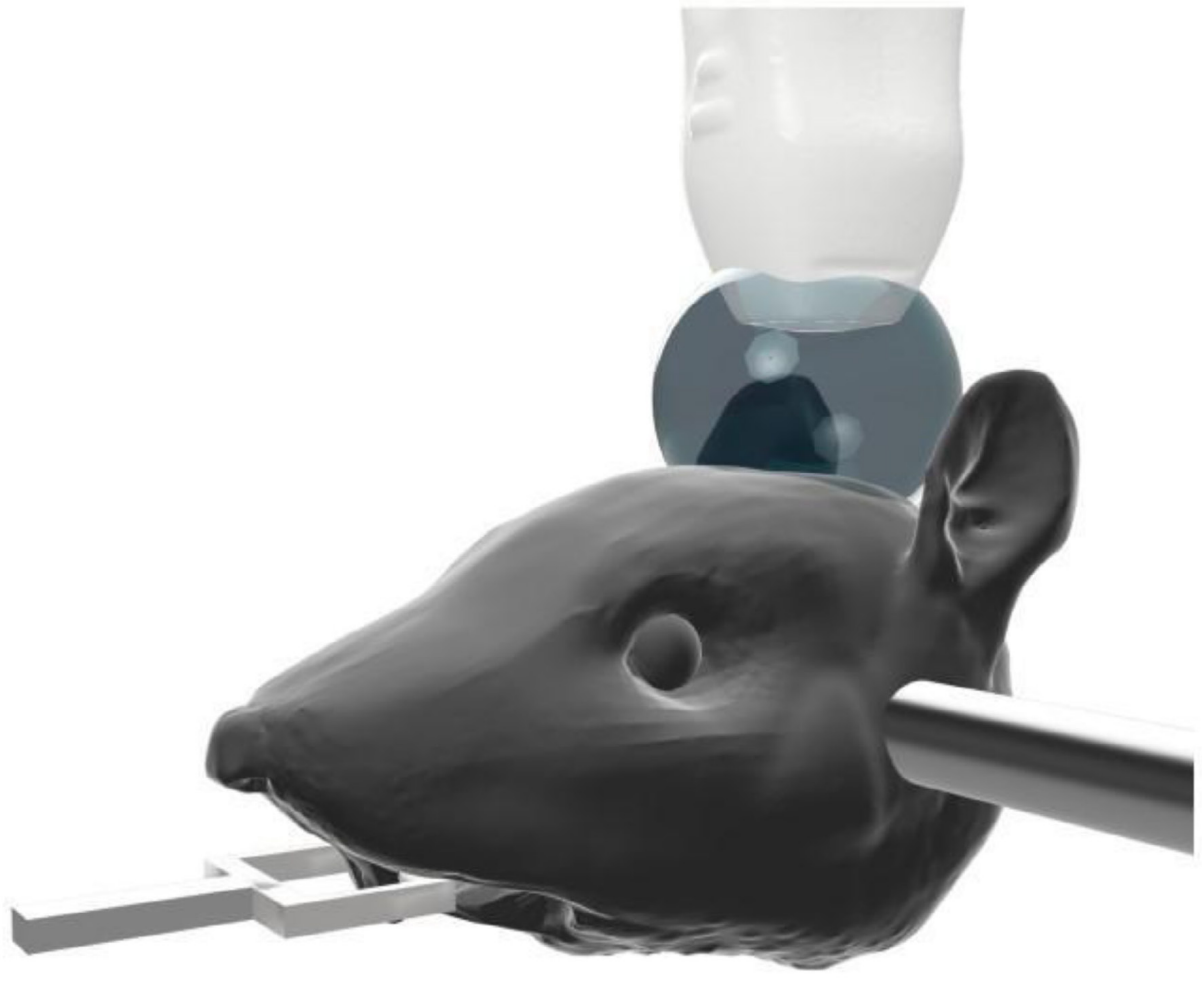
** The rat was anesthetized, and its head was fixed in the stereotactic frame.** Its head was stabilized using the ear bars. The tips of the ear bars were covered with a 3-D printed tip to mitigate any reflections that would otherwise echo from the dense metal bars. Echographic gel was placed atop the skull, and the ultrasonic probe was positioned 10 mm off the surface of the skull to avoid artifacts from skull reverberation in the images.

A Fischer 344 3-4-week-old female rat (Charles River Laboratories, Durham, NC) was prepared in accordance with protocols established with the Institutional Animal Care and Use Committee at the University of North Carolina at Chapel Hill. The animal was anesthetized using isoflurane gas carried by medical air, which has been shown to result in longer contrast circulation time compared the use of oxygen 44. The weight of the animal was measured and used to formulate the diluted solution of MB contrast. A catheter was inserted into the tail vein, and the MB dilution was infused using a syringe pump (Harvard Apparatus, Holliston, MA). The hair on the head was removed via a razor and a chemical hair removal solution to prevent acoustic artifacts in the ultrasound images, but otherwise the animal's skin and skull was not disturbed. The animal was then placed on a heating pad (FUJIFILM Visualsonics Inc., Toronto, ON, Canada), and a heat lamp was used to maintain body temperature and avoid temperature-induced vascular effects. The head was fixed in a stereotactic frame (Stoelting Co., Wood Dale, IL) and covered with echographic gel to couple it to the transducer, as shown in Figure 1. While maintaining about a 10 mm standoff between the transducer and the surface of the animal's head results in an increased amount of data to save with each acquisition, the standoff is necessary in order to remove a reverberation clutter artifact produced by the skull. In total, this imaging method was applied on a total of 5 rats with similar results among all animals.

We utilized a 1024 volumetric ultrasound system composed of four Verasonics Vantage systems operated by four controllers, which provided the ability to operate all channels simultaneously. We designed an ultrafast ultrasound sequence consisting of a 5-angle plane-wave compounding scheme (-3^◦^,0^◦^, +3^◦^ in the lateral and elevation dimensions) with a one-cycle transmitted waveform using 67% duty cycle with a center frequency of 7.81 MHz. With this sequence, we were able to acquire the radio frequency (RF) data continuously at a rate of 500 volumes per second (vps).

To image a volumetric region, we used the 32x32 Vermon (Vermon S.A., Tours, France) matrix array. This probe features a 7.81 MHz center frequency with a 60% bandwidth. The element pitch is 0.3 mm. We chose to drive the probe with a 20 V emission, which corresponds to a mechanical index (MI) of about 0.64, according to measurements made with an Onda (Onda Corporation, Sunnyvale, CA, USA) hydrophone in water. It should be noted that the MI reported here has not been de-rated to account for the attenuation of the skull.

**Figure 2:**
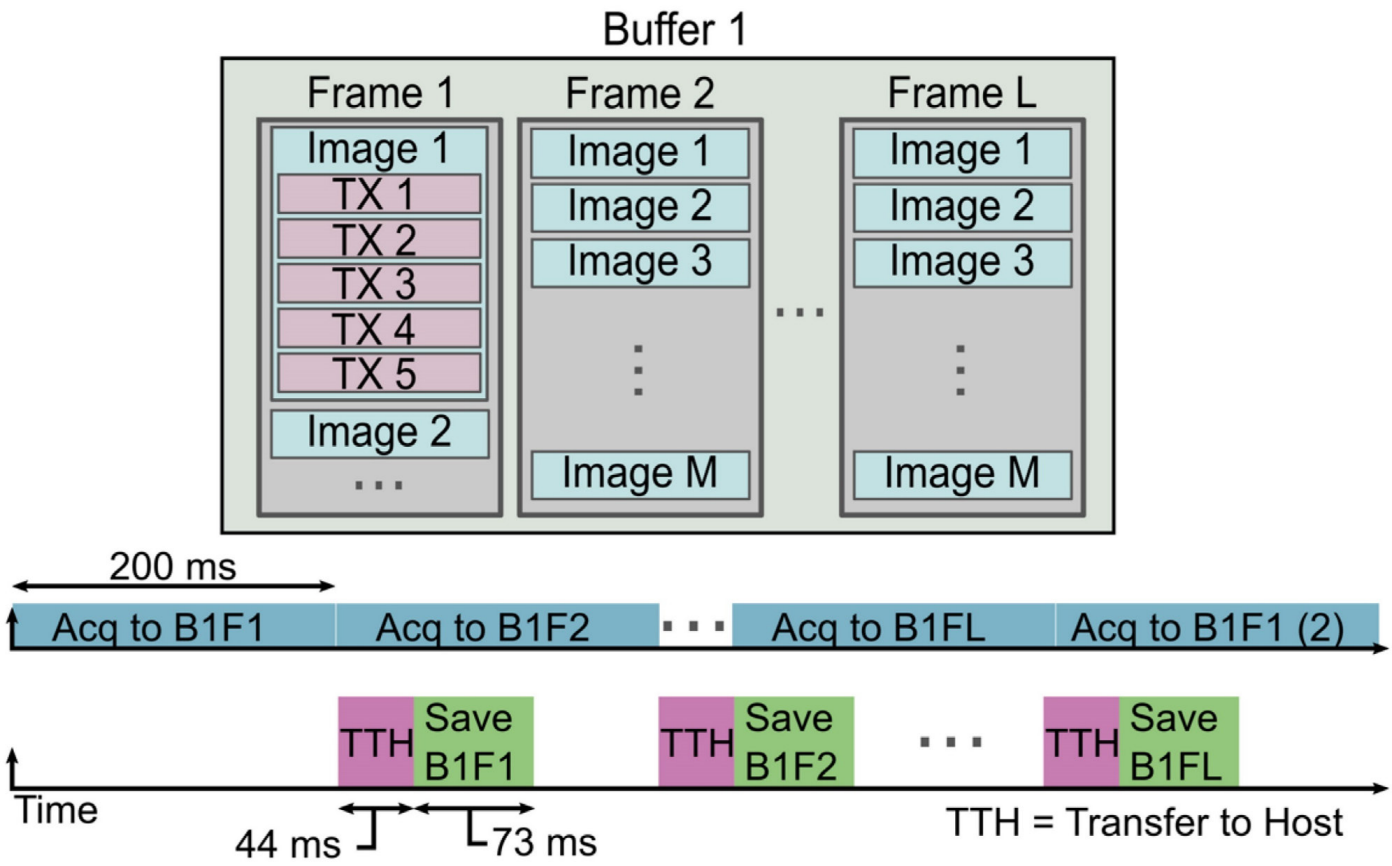
** The asynchronous acquisition, or “streaming,” is a process of collecting and saving data at such a high rate that there are no interrupts in the data collection.** This process begins by acquiring the data to the first frame of the buffer. When enough data has been collected to fill the pre-allocated size of the frame, the system initiates a transfer to host and saves the received data to a local SSD. Simultaneously, the Vantage system begins acquiring data to the next frame of the buffer. This process repeats until all frames of the buffer have been filled and until their saving processes have been initiated. The Vantage software and hardware loops back to reuse the first frame for the continued data acquisition. By this point, all data that was acquired to frame 1 has been saved, and the frame is ready to receive new data. The Vantage software and hardware continues to loop over the frames for the duration of the acquisition until the user-specified acquisition time has been reached.

This process of streaming data is accomplished by utilizing a single buffer and L frames on the Vantage system, as shown in Figure 2. The choice of the number of buffers and frames is variable and can be determined by the user. For instance, we chose to allocate 1 buffer and 8 frames in that buffer for writing data to the system. However, it is possible to use multiple buffers and a single frame per buffer, or some combination of multiple buffers and multiple frames, if desired. The user also defines a number of acquisitions to acquire to each frame of the buffer. These acquisitions may consist of a variable number of transmits needed to reconstruct a variable number of B-mode images. In our case, we allocated memory for each frame to contain 100 images' worth of 5-angle coherently compounded plane-wave data, which we define as a block. Therefore, we acquired a total of 500 acquisitions to each frame. The user must be careful when choosing the number and size of the frames and buffers since there is a fixed size limit, as described in the Verasonics Vantage documentation.

When the system begins the acquisition, the RF frames are written to the first frame in the buffer. Once the pre-allocated amount of data has been written to the frame, the system moves to the next frame while asynchronously transferring the data in the first frame to the host and saving that data to the drive. Once the system has reached the last frame in the buffer, the Vantage software/hardware loops back to use the first frame in the buffer, which has been cleared and is ready to receive new data. This imaging approach enables us to acquire data at a constant acquisition rate over long scan times. We collected a total of 200 s of data, which corresponds to 100,000 total volumes. The exact acquisition and streaming parameters used for our experiment are shown in Table 1. It is important to keep in mind that data transfer and saving rates can vary and are not static. The values represented here are average values recorded for the given data block size.

The binary files containing the acquisition data were transferred to another computer for offline beamforming and processing. The data was beamformed using delay-and-sum beamforming on a *λ/*2 (98.6 *µ*m) isotropic beamforming grid to improve the processing speed. Each beamformed volume covered physical space that was 9.5 × 9 × 10 mm^3^ in the axial, lateral, and elevation dimensions, respectively. The beamforming process was accelerated using four Nvidia (Nvidia Corporation, Santa Clara, CA) RTX-3090 graphics processing units (GPU) to reduce the beamforming time to a total of 8 hours. The individually beamformed plane-wave data were compounded and saved to binary files in batches of 200 subsequent volumetric images. These batches of B-mode images were then loaded into MATLAB (The Mathworks, Inc., Natick, MA) and filtered using singular-value decomposition (SVD) filtering to isolate MB signal. Fifteen to twenty percent of the largest singular values were removed to reconstruct the MB signal. The envelope-detected SVD-filtered data was then normalized and thresholded using an empirically determined parameter (typically around 1% to 7% of the maximum image intensity) to remove low-intensity noise. The MBs were then localized using a sub-pixel resolution weighted centroid approach, which consists of the following steps. First, the envelope-detected SVD filtered data is convolved with a 3-D Gaussian kernel calibrated to the size of the point spread function (PSF) of the imaging system. This is followed by an empirically-determined threshold (typically around 1-4 standard deviations above the mean image intensity) that segments remaining blobs into distinct image components. The weighted centroid of each distinct blob within the volume is calculated using the MATLAB function *imregionalprops3*. The localized MBs were then tracked using the Hungarian algorithm (*simpletracker*) 45 and smoothed using a Savitzky-Golay filter (MATLAB *sgolayfilt*). Tracks below a total number of ten positions were discarded. The remaining sub-pixel MB tracks were then projected onto an isotropic *λ/*20 (9.8 *µ*m) pixel-size grid. The resulting super-resolved volume was Gaussian filtered (MATLAB *imgaussfilt3* with a standard deviation of 0.8 to slightly improve the visual appearance. Due to a thresholding limitation of the chosen localization algorithm, the same dataset was processed twice using the same localization algorithm but varying the choices of thresholding parameters used for the segmentation step of the localization algorithm. One was chosen such that low-intensity MB signal was not discarded before the weighted centroid calculation step. However, the choice of a low threshold deters the separation of closely-spaced high-intensity MB signal, which results in an inaccurate localization or no localization at all for the MBs in this densely packed region. To super-resolve regions of the volume where high-intensity MBs were densely spaced, the data was reprocessed with a higher threshold that resulted in the separability of the MBs before the calculation of the weighted centroid of each blob in the volume. The result of these two independent localization processes is two rendered volumetric images. These images were then combined by taking the average of all overlapped non-zero pixels in each image. This prevents vessels that were detected by both localization steps from being rendered with a much higher intensity compared to vessels that were only rendered with one of the localization steps.

We characterized the blood flow dynamics by measuring the velocity in individual MB tracks. The track velocities were determined by calculating the distance between consecutive points in a track and multiplying by the frame-rate and the physical pixel size. All pixels in the track were cumulatively assigned the velocity value associated with each section of the track. The total volume was then divided by a density map, so the effective average velocity was calculated at every point in the image. The volume containing the axial velocity data was median-filtered before plotting to remove remaining noisy tracks.

The resolution of the super-resolved volume was determined using the Fourier shell correlation (FSC) 39, which determines the resolution threshold of two independent reconstructions of the same image at which both reconstructions are consistent. We computed the FSC of our volume by first projecting the sub-pixel MB tracks onto a volumetric image with a pixel size of *λ*/20 (9.8 *µ*m). The single super-resolution volume was then split into four sub-images, the FSC was computed for each of the two pairs of sub-images, and then the resulting FSCs from both images were averaged together. This process is described in greater detail by Koho et al. 40.

All experimental setup volume renderings were created using Blender (Blender Foundation, Amsterdam, Netherlands) and Fusion 360 (Autodesk, Inc., San Rafael, CA). The 3-D ULM images were all rendered using the maximum intensity projection mode in 3D Slicer 46. The directional velocity volume was rendered using the volume containing the axial velocity information and its inversion in the maximum intensity projection mode in 3-D Slicer. This enabled us to render the vessels with opposing direction of flow with two separate color maps.

## Results

**Figure 3:**
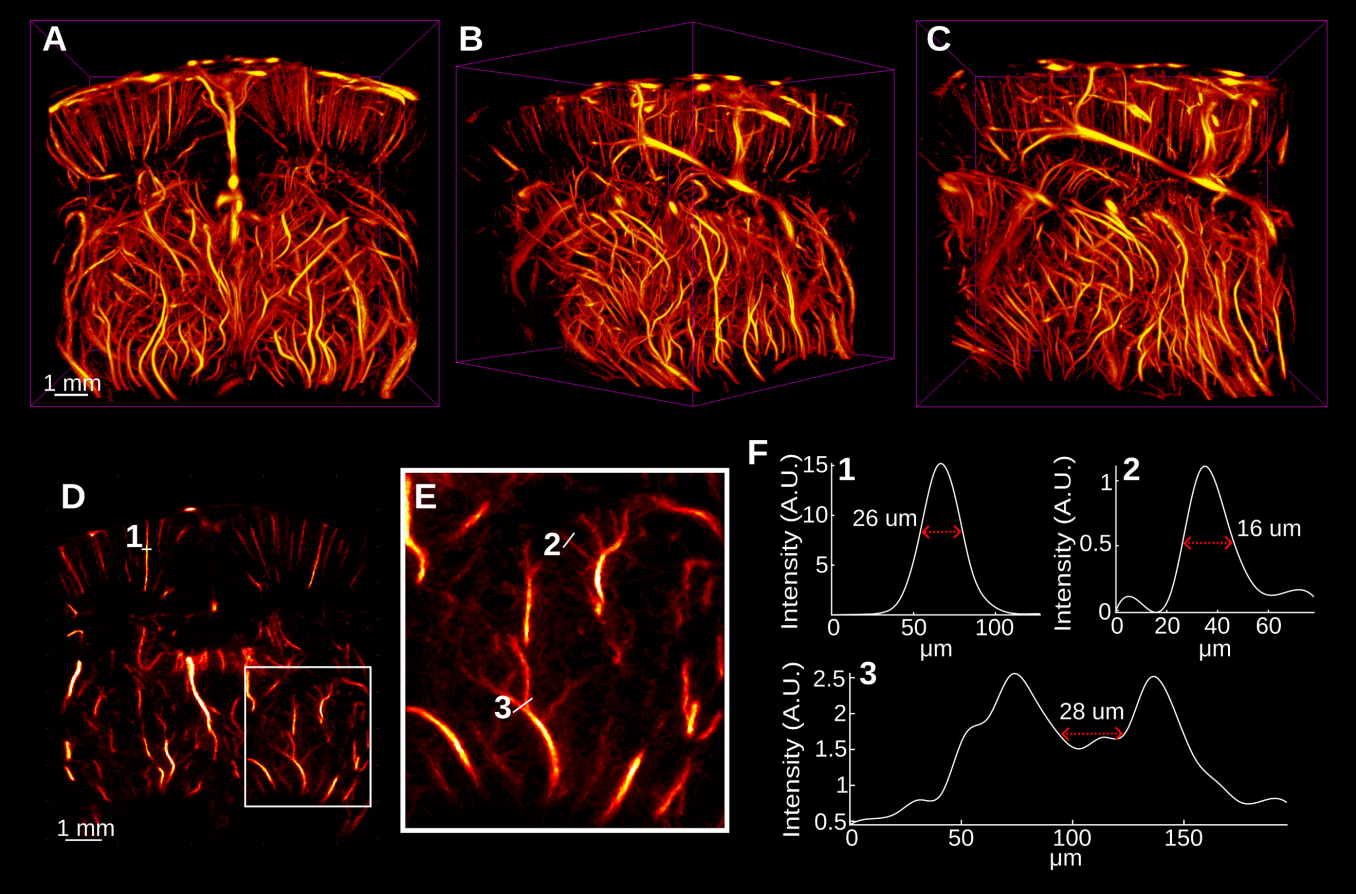
** A-C demonstrate the super-resolved rat brain vasculature that was acquired surgically noninvasively in three orientations. D Demonstrates a 392 *µ*m coronal section of the brain located approximately -2.9 mm from the anterior-most region of the image.** The white box in E demonstrates the vascular detail in an enlarged view. A few vessels were selected and the width of the vessels, as measured by the full-width half-maximum of the spline-interpolated cross-sections, are shown in E. We observed that vessels down to 16 *µ*m were clearly distinguished from background noise. We also observed that the vessels at a bifurcation were clearly distinguished up to 28 *µ*m by analyzing the minimum threshold intensity required to separate the vessels in F3.

The accumulation and tracking of all localized MBs during the acquisition were used to reconstruct the rat brain vasculature shown in Figure 3. Figure 3A-C shows the volumetric ULM image in various orientations, including the coronal and sagittal views. This volume was reconstructed using a total of 4.02 million bubble localizations. A 394 *µ*m section of the brain located -3 mm from the anterior of the volume is shown in Figure 3D. A zoomed in view of the section in Figure 3E demonstrates the fine vascular detail with vessels sized using a fullwidth half-maximum (FWHM) measurement down to 16 *µ*m. The separation at the onset of a bifurcation can be distinguished down to 28 *µ*m.

**Figure 4:**
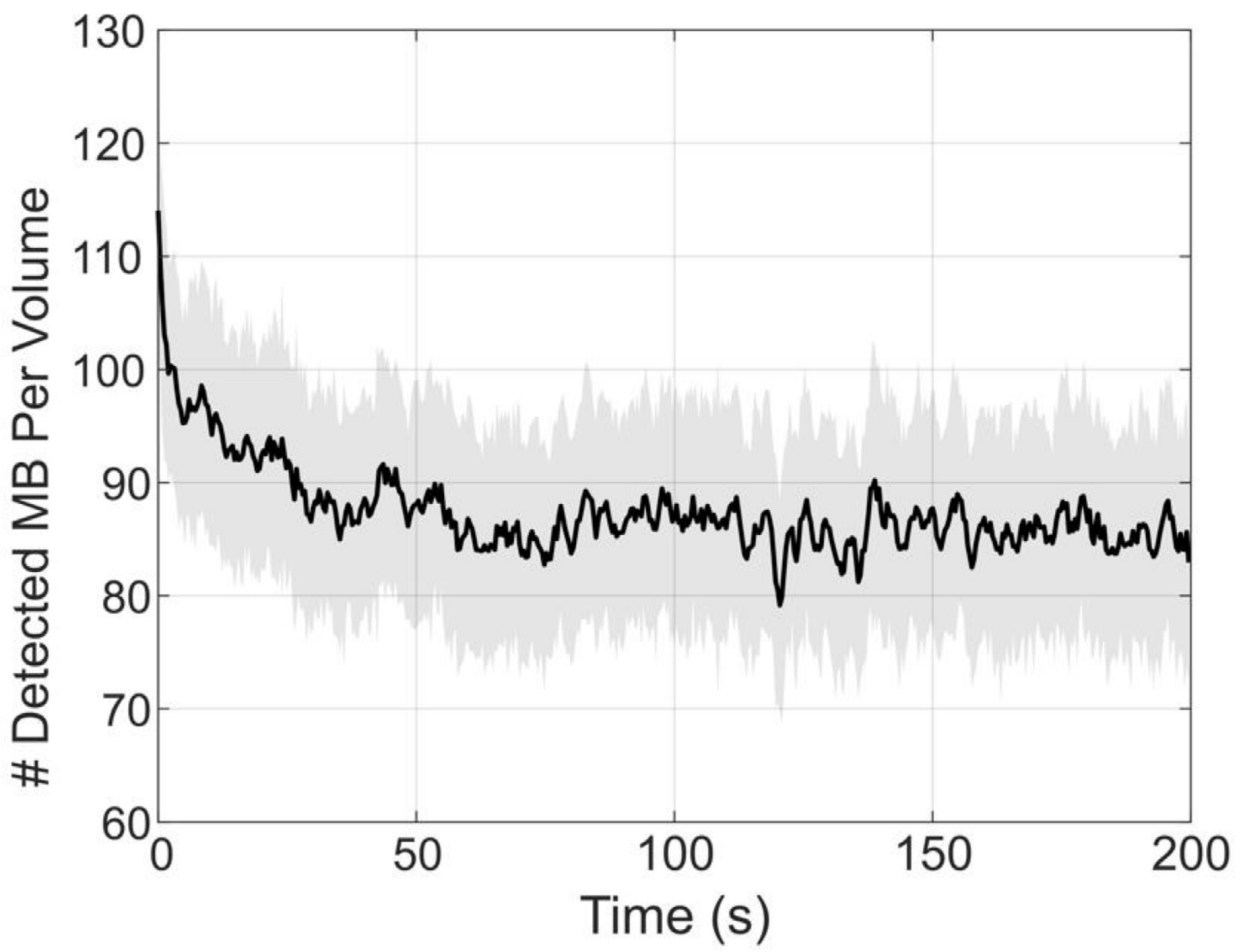
** The number of bubbles detected in each volume were counted and plotted over time to demonstrate the contrast behavior for the duration of the infusion. Each data point represents the mean of the number of bubbles detected over 200 frames (0.2 s).** The solid black line represents the box-filtered average using a window of 5 points (1 s) worth of data. The shade shows the standard deviation of the 200 frames used to compute the mean. The peak at the beginning of the data demonstrates a small 5 *µ*L bolus injected prior to the start of the acquisition, which is used to observe the contrast wash-in in live imaging. Over time, the number of detected bubbles per frame appears to reach a steady-state around 86 ± 3 bubbles localized per volume.

Figure 4 demonstrates the number of bubbles localized per volume through the duration of the experiment. From this plot, we can see an increased number of bubbles localized at the beginning of the collection, which was immediately started following a small bolus and the beginning of the infusion. This explains the asymptotic behavior of the number of detected bubbles. After about 35 to 50 seconds, the concentration of bubbles in the blood reaches steady-state with an average of 86 ± 3 bubbles localized per volume.

**Figure 5:**
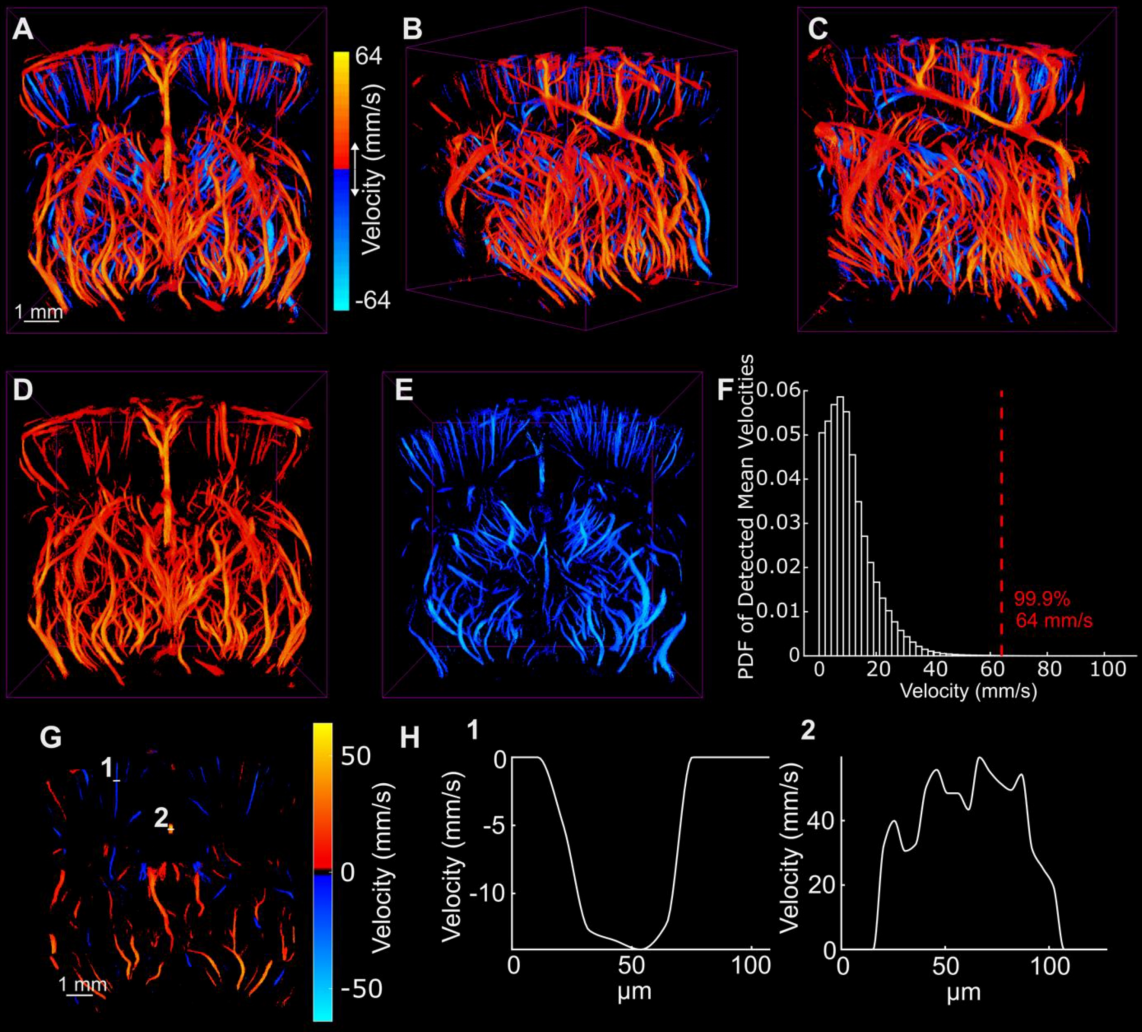
A-C show the flow in the axial direction with the same orientations shown in Figure 3. D and E demonstrate the isolated positive and negative velocity shown in A. F illustrates the probability density function of the detected velocities everywhere in the volume and illustrates a 99.9 percentile cutoff used for image saturation. G demonstrates the axial directional flow in the same section shown in Figure 3D. Two cross sections shown in G were plotted in H to demonstrate the velocity in a small cortical vessel and a large vessel that runs through the center of the brain.

Figure 5 demonstrates the directional blood flow in the axial dimension. As mentioned previously, there were many noisy tracks that were removed using median filtering to render these images. The histogram of the magnitude of the axial velocity detected in the volume was computed to determine an upper bound at which to set the saturation of the velocity color map. This histogram is shown in Figure 5F. The upper bound was chosen such that 99.9% of detected velocity magnitudes were represented in the data. This limit corresponded to about 64 mm/s. Extra care should be taken when reconstructing and representing vascular flow information in the vessels. This will be discussed in the following section.

While Figure 5A-C demonstrate both the positive and negative axial flow in the volume, Figure 5D-E demonstrate the isolated vessels with positive and negative flow, respectively. Figure 5G demonstrates a minimum and maximum intensity projection of the vessels in the same section shown in Figure 3D. The cross sections of a small vessel and a large vessel are shown in Figure 5H to demonstrate the flow profile across the vessel. In general, laminar flow is observed across both vessels. However, the large vessel shows a noisy parabolic shape that is most likely due to taking the cross-section of a MIP.

**Figure 6:**
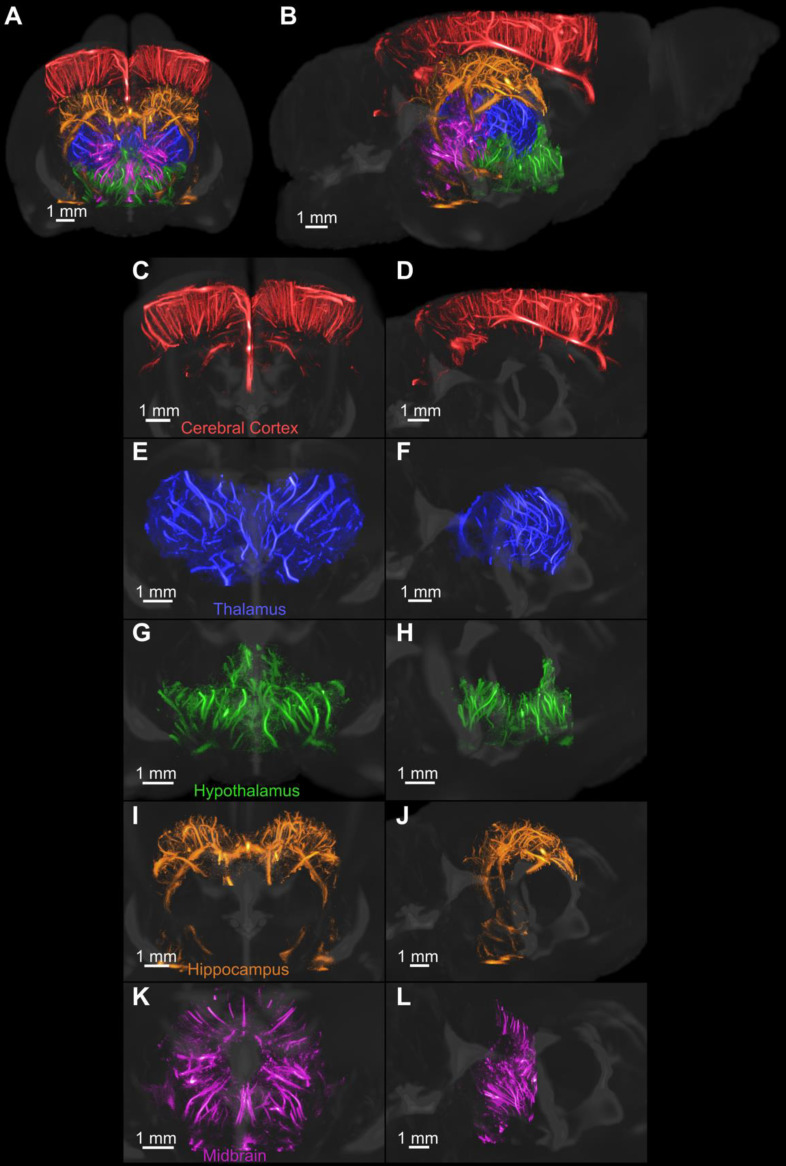
** The MRI-derived neuroanatomical rat brain atlas was used to segment five regions of the brain after manual registration.** Each region was isolated and rendered using a different colormap. All regions were then rendered together with an MRI scan that was provided with the atlas, as shown in A and B. The coronal and sagittal views of each region, including the C-D cerebral cortex, E-F thalamus, G-H hypothalamus, I-J hippocampus, and K-L midbrain, were rendered separately to demonstrate the vascular detail.

The volumetric super-resolution image was manually registered with a magnetic resonance image (MRI)derived neuroanatomical atlas of an adult Fischer 344 rat 41. A few regions of the brain were segmented, colorcoded and rendered with the provided 3-D MRI of the brain, as shown in Figure 6. Figure 6C-D demonstrates an isolated 3-D rendering of each of the selected regions of the brain to demonstrate the vascular detail. The colorcoded rendering improves the visual representation of the brain topography compared to the use of a single-color map for all vessels.

**Figure 7:**
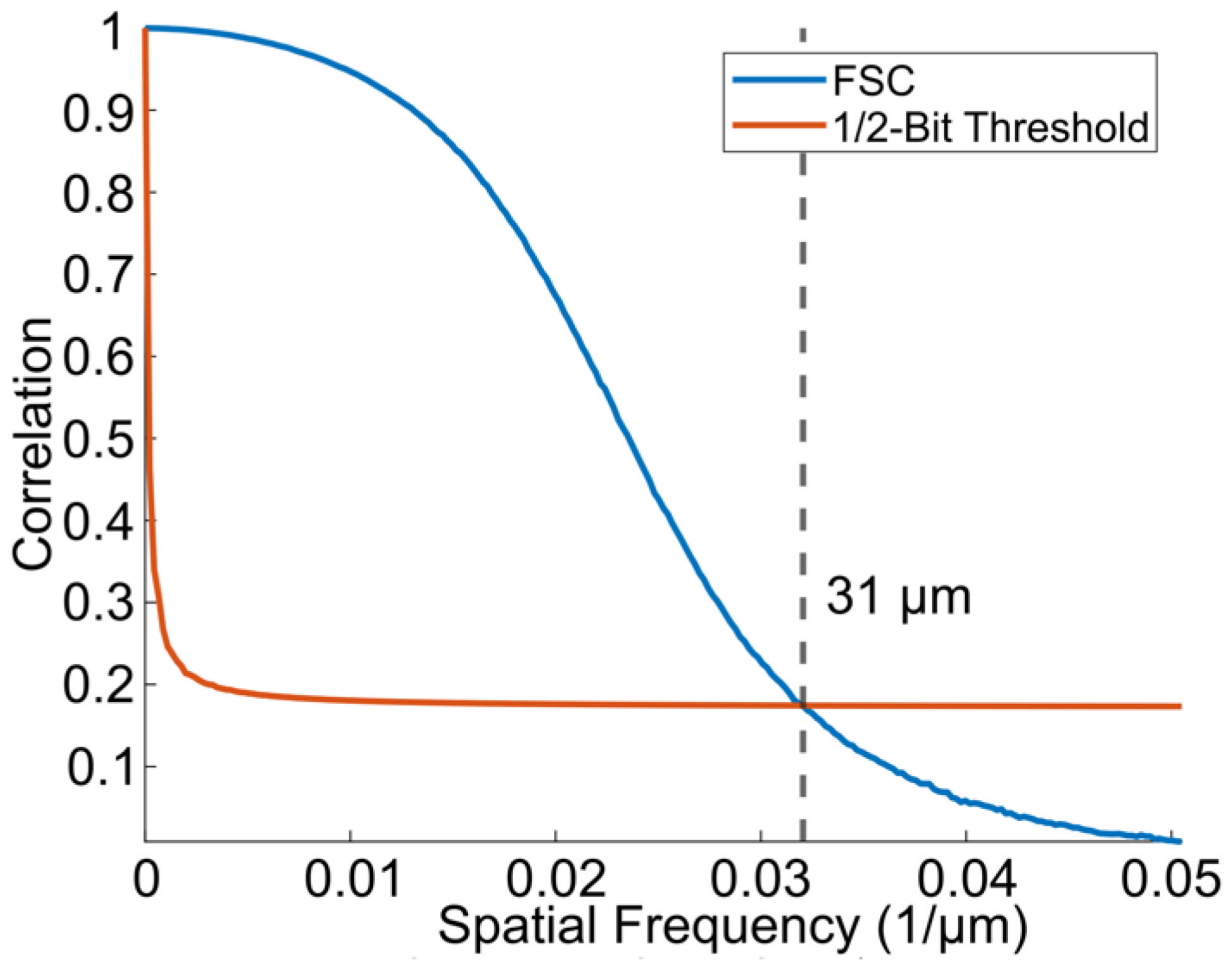
The resolution of the super-resolved image was determined using the FSC with a 1/2-bit threshold. The FSC yielded a resolution of 31 *µ*m, which is approximately 3 times the physical pixel size.

The results of the Fourier shell correlation, shown in Figure 7, demonstrate a resolution of 31 *µ*m, which is an improvement in resolution by a factor of 3.1 in the axial dimension and roughly 9.5 in the lateral and elevation dimensions compared to the resolution of conventional B-mode ultrasound imaging (which is *λ/*2 and 1*.*5*λ*, respectively).

## Discussion

We implemented a high frame-rate volumetric acquisition system with continuous data streaming that enables a constant frame rate for the entirety of the acquisition. As a result, the system was able to acquire the same amount of data in a much shorter amount of time compared to methods that require pausing for data transfer. This has significant ramifications for acquiring data in a clinical setting since lower scan times reduce patient stress and improves the utilization efficiency of the imaging technology in the clinic. As mentioned previously, Huang et al. have already shown the ability to fully resolve vasculature in humans using datasets that were acquired in a matter of seconds in 2-D 33. Increasing the number of frames acquired in a short time is likely to improve the quality of the super-resolution image.

A constant frame-rate is advantageous for motion tracking in high motion environments, such as in clinical imaging and in implementing filters, including the SVD filter, which benefit from long persistence lengths in slow time. If there are breaks in acquisition, the data collected after such a movement could become useless to the data collected in the original position since motion tracking at sub-resolution volumes relies on speckle realizations that are not completely independent. Constant acquisition has the ability to compensate for such a scenario by providing complete information about the imaging target throughout the duration of the acquisition.

The streaming method requires that reconstruction is postponed, resulting in the fast saving of RF data. Saving the RF data has the benefit of beamforming with versatility. Optimization of the beamforming parameters or approaches, including, apodization, plane wave masking, F-number restrictions, phase coherence factor 47 can then be used. This could provide better data processing, since parameters can be tuned iteratively to optimize the beamforming of each dataset.

We used a 3-D acquisition system that consisted of four Verasonics Vantages, which enabled synchronous acquisition of data across all 1024 data channels. The utility of this system is important for acquiring truly 4-D data in all positions in the field of view (FOV). It also reduces the number of required acquisitions to reconstruct one image, which is a limitation of multiplexing. With this system, it is possible to use more plane-waves to improve the image quality while maintaining a fast imaging rate. While from one point of view, programming these machines may be simpler since the headache of multiplexed acquisition is avoided, we initially experienced some difficulties with programming the acquisition such that each of the four systems acquired synchronously without failure. The system also requires significantly more hardware that decreases the portability of the system.

Beamforming in 3-D, however, poses a significant computational burden that may require days for beamforming, depending on the physical size of the volume, the beamforming grid density, and the number of frames. We ameliorated this issue by compiling algorithms for parallel beamforming across four Nvidia RTX 3090 GPUs. This tremendously reduced the required beamforming time down to a total length of about eight hours for 100k volumes. Without GPU-accelerated image reconstruction, it is arguable that offline reconstruction is too impractical to be useful since the number of frames required to create a super-resolution image is very high. When optimizing the microbubble concentration, we observed that higher concentrations tended to provide increased microbubble localizations in small vessels while decreasing microbubble separability in large vessels. It was more difficult to resolve large vessels as a result. One solution to this problem has been demonstrated by Huang et al., who showed a microbubble separation technique that is based on their spatiotemporal signatures 31.

One of the intended uses of ULM is to detect and characterize disease by analyzing the vascular flow dynamics. There have been many papers that have published astounding images of vascular flow with velocities that appear to be consistent with literature. It's important to keep in mind, however, that these reported values can be easily skewed by bubble tracking parameter selection. The selected maximally-allowed pairing distance parameter directly affects the upper bound of the range of detectable bubble velocities. This means that the reported velocities in a super-resolution velocity map are subject to user-dependent parameter. By allowing bubbles to track at too short a distance, the maximum detected velocity may be lower than the real speed of blood flow in the vessels. The drawback of allowing an increased pairing distance is that more noisy microbubble pairings will be made. The latter problem can be ameliorated by throwing out tracks below a particular length, but this is also at the cost of resolving small vessels that may have a low number of MB events. Further filtering by removing tracks that exceed a particular turning angle or acceleration could help, but these parameters are also subjective. The danger is that such filtering could destroy vascular abnormalities, such as tortuosity, decreasing the detection sensitivity for many of the aforementioned diseases. The researcher should remember to keep these points in mind when choosing image processing parameters.

In this work, we found that it was necessary to combine two iterations of the localization step with different segmentation threshold parameters (one low and one high) in order to resolve more vasculature. This was due to an inadequacy in the localization algorithm that depends on uniform bubble brightness throughout the image. This is often not the case, however, in complex imaging environments. In this case, many bubbles had a low intensity that required a low segmentation threshold in order to localize. This was the first approach we took to resolve the vasculature. However, we discovered that regions of the image with a high density of bright bubbles were not segmented properly, and as a result, were empty on the final ULM rendering. To fill in these regions, we processed the entire dataset again with a higher segmentation threshold and combined the result with the first ULM result to achieve a more populated vascular map. This illustrates the inadequacy of a global threshold for bubble segmentation in this algorithm. This step could be improved by implementing a spatially local threshold to improve segmentation of bubbles across a wide range of intensities.

ULM was used to reconstruct rat brain vasculature in three dimensions with resolutions up to 9 times that of conventional ultrasound imaging. The capabilities of this microvasculature imaging modality have been well-known for several years and this method has been demonstrated repeatedly *in vivo* 28-30,36,37 and has been translating to the clinic in recent years 33. The potential utility of this method is vast, including the detection of cancer 48, the detection of aneurisms 32, and other diseases by reconstructing and analyzing vessel geometry and flow characteristics.

We have not yet seen the translation of 3-D ULM into the clinic. However, its advantages of enhanced vessel characterization and motion tracking due to the third dimension poise the method for potentially increased diagnostic power. Some limitations, however, will need to be addressed to improve the versatility of this method. The roughly 1 cm^2^ size of the probe used for this research is great for small targets in animals, but the field of view significantly restricts its usefulness for imaging entire human organs. Some sort of translation scheme with overlapping areas, such as that shown by Heiles et al. 49, could be applied. However, it may be more useful to design transducers with a larger spatial footprint using a density-tapered sparse array design 50,51, for example.

## Conclusions

We demonstrated the development of a continuous ultrasound image acquisition sequence at a fixed high frame-rate that significantly reduces the required scan time for volumetric ULM reconstruction. We reconstructed a volumetric ULM image acquired in a completely surgically non-invasive way through the skull, and we demonstrate a FSC resolution of 31 *µ*m, which is nearly ten times the resolution of conventional ultrasound imaging. We demonstrated the sensitivity of this method for the resolution of directional blood flow in the microvasculature. We also isolated distinct regions of the brain by registering the rat brain vasculature with a neuroanatomical atlas and utilized an array of color-codings to improve topographical visualization of the brain. The results of our non-invasive, transcranial imaging demonstrate the utility of this ultrasonic imaging modality for longitudinal studies of the brain in rodents.

## Figures and Tables

**Table 1:** Data Acquisition and Saving Parameters

Number of samples/channel/acquisition	1024
Number of TX/acquisition	5
Number of frames/acquisition block	100
Data size/Vantage/acquisition	245 MB
Total data size/acquisition	980 MB
Total dataset size	980 GB
Save disk type	2 NVMe drives forming a raid0
Block acquisition time	200 ms
Block transfer time	~44 ms
Block saving time	~73 ms
